# Chromosome 7q31 POAG locus: ocular expression of caveolins and lack of association with POAG in a US cohort

**Published:** 2011-02-08

**Authors:** Markus H. Kuehn, Kai Wang, Ben Roos, Edwin M. Stone, Young H. Kwon, Wallace L.M. Alward, Robert F. Mullins, John H. Fingert

**Affiliations:** 1 Department of Ophthalmology and Visual Sciences, Carver College of Medicine, University of Iowa, Iowa City, IA; 2 Department of Biostatistics, University of Iowa, Iowa City, IA; 3 Howard Hughes Medical Institute, Iowa City, IA

## Abstract

**Purpose:**

To determine the role of the recently discovered primary open angle glaucoma (POAG) risk factor mapped to chromosome 7q31 in glaucoma patients from Iowa and to determine the expression pattern of genes in the locus in human eyes.

**Methods:**

A cohort of 545 POAG patients and 297 control subjects from Iowa were genotyped with a single nucleotide polymorphism (SNP; rs4236601) in the chromosome 7q31 locus using a quantitative polymerase chain reaction (PCR) assay. The expression of genes within the 7q31 locus, caveolin-1 (*CAV1*) and caveolin-2 (*CAV2*) in human eyes was investigated with immunohistochemistry.

**Results:**

The minor allele frequency (MAF) of rs4236601 was 27% in control subjects and 29% in POAG patients. We detected no statistical difference when we compared the allele frequencies of rs4236601 between POAG patients and control subjects (p=0.5). Similarly, we detected no statistical difference in the frequency of the three possible rs4236601 genotypes between patients and controls (p=0.22). Immunohistochemistry showed caveolin expression in human retina, ciliary muscle, trabecular meshwork, and Schlemm’s canal. In our small cohort of donor eyes, the genotype of rs4236601 did not obviously influence labeling intensity or distribution of CAV1 and CAV2 in the retina.

**Conclusions:**

A genome-wide association study of subjects from Iceland mapped the first common genetic risk factor for POAG to a small region of the genome on chromosome 7q31 that contains the caveolin genes *CAV1* and *CAV2*. We were unable to detect this association in our patients from Iowa, suggesting that this risk factor may not have a strong effect in all populations.

## Introduction

The glaucomas are a group of eye diseases that primarily affect the optic nerve and retinal ganglion cells and are a common cause of vision loss and blindness. Current estimates suggest that over 60 million people have glaucoma worldwide [1]. Glaucoma is defined by progressive cupping of the optic disc and a corresponding loss of visual field. Several risk factors for glaucoma have been identified including age, race, family history, intraocular pressure (IOP), and corneal thickness. The most common form of glaucoma in the United States is primary open angle glaucoma (POAG) [2]. A subset of POAG that occurs with a maximum IOP below an arbitrarily threshold of 21 mmHg is often termed normal tension glaucoma (NTG).

Genetic variants have also been shown to have a significant role in the pathogenesis of POAG. Studies of rare families have mapped the position of 14 POAG genes to small regions of the genome [3-6] and glaucoma-causing genes have been discovered in two of these loci, myocilin (*MYOC*) [7] and optineurin (*OPTN*) [8]. Mutations in *MYOC* are associated with POAG that is characterized by high IOP and strong family history and are responsible for approximately 4% of POAG cases worldwide. Mutations appear to cause myocilin protein to become misfolded which leads to increases in outflow resistance, elevated IOP, and subsequently the development of POAG [9]. Mutations in *OPTN* have been associated with rare cases of NTG [8,10]. The protein encoded by *OPTN* is involved in many biologic processes including some that lead to apoptosis [11,12], however, the mechanism by which mutations in this gene cause glaucoma is unknown. An additional gene, WD repeat domain 36 (*WDR36*), was identified by family-based studies. However, its role in glaucoma pathogenesis is controversial [13-17]. These studies have identified high-penetrance mutations that cause glaucoma in a relatively small proportion of POAG cases. Most carriers of *OPTN* and *MYOC* mutations develop glaucoma and asymptomatic carriers are rare.

Recently, a population-based study of glaucoma identified the first common genetic risk factor for POAG. A genome-wide association study of 1,263 POAG subjects and 34,877 control subjects from Iceland by Thorleifsson and coworkers [18] identified a region of the genome on chromosome 7q31 that is associated with glaucoma. The specific DNA variation that confers risk for POAG in this locus is unknown. Although, the chromosome 7q31 locus spans the caveolin 1 (*CAV1*) and caveolin 2 (*CAV2*) genes, no DNA variations in these genes were found to be associated with POAG. However, the minor allele of one genetic marker, rs4236601, was tightly associated with POAG with a p=5.0×10^−10^. When Thorleifsson and coworkers [18] tested a second cohort of 4,239 POAG patients and controls from Sweden, the UK, and Australia with the rs4236601 marker, the association with glaucoma was confirmed but was statistically much weaker (p=0.0015). When a third cohort of POAG patients and controls from Hong Kong and Shantou China were studied, the association between rs4236601 and glaucoma was again confirmed with similar statistical significance (p=0.003) [18]. The role of the POAG risk allele at the chromosome 7q31 locus appears to have significance that varies between populations. Consequently, we set out to investigate the importance of this glaucoma risk factor by genotyping our cohort of POAG patients and control subjects from Iowa at the rs4236601 marker. We also examined the expression of the two genes in the chromosome 7q31 locus (*CAV1* and *CAV2*) using an immunohistochemistical study of human donor eyes.

## Methods

The study was approved by the University of Iowa’s Institutional Review Board and informed consent was obtained from study participants.

A cohort of 545 subjects were examined by fellowship-trained glaucoma specialists and received complete ophthalmic examinations (including gonioscopy, standardized visual field testing, and stereoscopic optic nerve examination). Subjects were diagnosed with open angle glaucoma when characteristic optic nerve damage and corresponding visual field defects were detected in at least one eye and the maximum IOP was greater than 21 mmHg as previously described [19]. A cohort of 297 unrelated normal subjects from Iowa that had no evidence of glaucomatous optic nerve damage and had IOP <22 mmHg were enrolled as control subjects. Normal subjects were not specifically evaluated for the presence of exfoliation syndrome. Blood samples were obtained from study participants and DNA was prepared using a non-organic method [20].

The power of this Iowa sample to detect a disease odds ratio of 1.6 is 87% at a significance level α=0.05, 70% at α=0.01. For a disease odds ratio of 1.8, it is 97% at α=0.05, 88% at α=0.01. For a disease odds ratio of 1.4, it is 62% at α=0.05, 38% at α=0.01. Power calculations were made using an online power calculator for for logistic regression with binary covariates. The value of MAF was set at 0.28, between the MAF in Iowa cases and in Iowa controls.

Subjects were genotyped at single nucleotide polymorphism (SNP) rs4236601 with a quantitative polymerase chain reaction (PCR) assay using the manufacturer’s protocol (TaqMan; Applied Biosystems, Carlsbad, CA). Genotype calls were confirmed by DNA sequencing in 10 subjects using standard conditions and primers that are available on request. The frequency of rs4236601 alleles and genotypes were compared between patients and controls using Fisher’s exact test and the software package R. A threshold p<0.05 was considered significant.

Retinal tissue from human eye donors without reported ocular diseases was obtained and preserved in 4% paraformaldehyde within 5 h postmortem. Following genotyping for rs4236601, six donors were chosen for immunohistochemical analysis, three of which were homozygous for each genotype. Tissue was embedded in optimum cutting temperature medium (OCT; Ted Pella, Inc., Redding, CA), frozen and sagittal sections at a thickness of 7 μm were obtained. Caveolin 1 was detected using a goat primary antibody (1:200 dilution; Abcam, Cambridge, MA) and a Cy3 conjugated secondary antibody (Invitrogen, Carlsbad, CA). Caveolin 2 was detected using a rabbit primary antibody (1:200 dilution; Abcam) and a Cy5 conjugated secondary antibody (Invitrogen). Images were counterstained with 4’-6-Diamidino-2-phenylindole (DAPI) for orientation. Negative controls included sections incubated with secondary antibody alone. The expression of caveolin 1 and caveolin 2 were each studied in the anterior segments of two donor eyes using the same immunohistochemistical methods.

## Results

A cohort of 545 POAG patients and 297 control subjects from Iowa was genotyped with the genetic marker, rs4236601, that was previously associated with POAG [18]. The size of this cohort has a 87% chance of detecting an association with POAG that has an odds ratio of 1.6 or greater with a p<0.05. Similarly, our study has 62% chance to detect an association with an odds ratio of 1.4 or greater (p<0.05), which is similar to the association detected by Thorleifsson et al. [18] in their cohort of Icelandic subjects.

The minor allele frequency of rs4236601 in our cohort from Iowa is 28.5% in the POAG patients and 26.9% in control subjects (Table 1). No significant difference was detected in allele frequency between the POAG patients and controls (p=0.50). Similarly the frequency of each of the three possible genotypes of rs4236601 were compared between POAG patients and controls (Table 1) and no significant difference was detected (p=0.22).

**Table 1:** Allele and genotype frequencies of SNP rs4236601 in POAG patients and control subjects from Iowa.

**Alleles**	**POAG (n=545)**	**NL (n=297)**	**Genotypes**	**POAG (n=545)**	**NL (n=297)**
G	779 (71.5%)	434 (73.1%)	G/G	274 (50%)	163 (55%)
A	311 (28.5%)	160 (26.9%)	A/G	231 (42%)	108 (36%)
			A/A	40 (7.3%)	26 (8.8%)
p-value=0.50			p-value=0.22		

**Figure 1:**
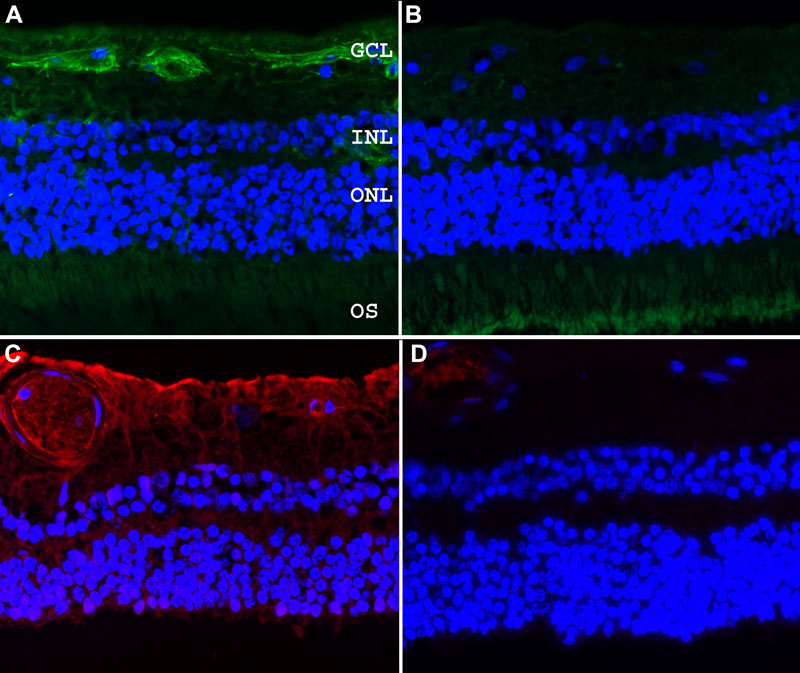
Caveolin 1 and 2 in the human retina. Immunohistochemical detection of Caveolin 1 (**A** and **B**) and Caveolin 2 (**C** and **D**) in the human retina. Negative controls with no primary antibody (**B** and **D**) revealed only minor cross reactivity of the secondary antibodies. GCL=ganglion cell layer, INL=Inner nuclear layer, ONL=outer nuclear layer, OS=Outer segments.

**Figure 2:**
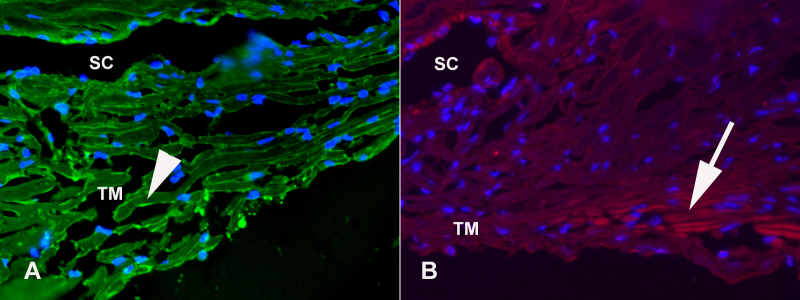
Caveolin 1 and 2 in the human iridocorneal angle. Immunohistochemical detection of Caveolin 1 (**A**) and Caveolin 2 (**B**) in the iridocorneal angle. Caveolin 1 reactivity was mainly associated with the trabecular meshwork cells lining the trabecular meshwork beams (Arrowhead) and Schlemm’s canal. Caveolin 2 is mainly detected in Schlemm’s canal and the longitudinal ciliary muscle. (SC: Schlemm’s canal, TM: Trabecular meshwork, Magnification: (**A**) 400×, (**B**) 200×).

Immunohistochemical analysis of human retinal sections from 6 donors indicate that Caveolin 1 is prominently localized in association with the ganglion cell layer (Figure 1A) Perivascular labeling of small and large retinal vessels, the nerve fiber layer and inner plexiform layer was also occasionally observed. Caveolin 2 immunoreactivity was most pronounced throughout the nerve fiber and ganglion cell layers (Figure 1C). Weak immunoreactivity was also observed in the inner and outer plexiform and nuclear layers, but not in association with photoreceptor inner and outer segments. A radial labeling pattern was evident in the inner plexiform layer, suggesting that Muller cells are at least in part responsible for Caveolin 2 expression in the retina. Retinal vascular elements were variably immunoreactive. We did not observe obvious qualitative differences in the labeling patterns and intensity when the retinas of donors homozygous for the minor and major alleles were compared to each other. Immunohistochemical analysis of the anterior segments of 2 donors demonstrated that Caveolin 1 and Caveolin 2 are both localized to Schlemm’s canal, while Caveolin 1 is also present in the trabecular meshwork (TM) and Caveolin 2 is also present in the ciliary muscle (Figure 2).

## Discussion

POAG is a heterogeneous collection of retinal ganglion cell and optic nerve diseases. Studies of rare, large POAG families have provided evidence for the existence of at least 14 genes that when defective or mutated can cause glaucoma without many contributions from other genes or the environment. Two of these high penetrance glaucoma genes, *MYOC* and *OPTN*, are responsible for around 4% of POAG cases overall. A significant fraction of the remaining cases of POAG are likely caused by the combined influence of mutations in many genes and the action of environmental factors. Such genetic factors may each contribute small risk for developing POAG on their own and will be frequently observed in subjects that never develop glaucoma. Thorleifsson et al. [18] recently mapped the first of these common genetic risk factors for POAG to a small region of chromosome 7q31 with a genome-wide association study of subjects from Iceland. The chromosome 7q31 locus is tagged by the minor allele of rs4236601 and contains two genes, *CAV1* and *CAV2*, although no glaucoma-associated mutations in these genes have been found to date [18]. The glaucoma risk factor that is in linkage disequilibrium with the minor allele of rs4236601 has not yet been discovered.

Given the recent association study results, *CAV1* and *CAV2* have been implicated in glaucoma pathogenesis. The family of caveolin genes encode proteins that form caveolae, invaginated plasma membrane structures that are believed to have an important role in the transport of macromolecules across membranes (transcytosis) and influence signaling pathways by regulating the positioning of signaling molecules in the membrane. Caveolin 1 and caveolin 2 are expressed in non-muscle cells while caveolin 3 is present in skeletal and some smooth muscle cells [21]. Although no glaucoma-causing mutations have been identified in either *CAV1* or *CAV2* to date, the results of the recent genome-wide association study suggests their existence. The mechanism by which defects in either *CAV1* or *CAV2* might cause glaucoma is unknown, but it has been hypothesized that such mutations might lead to glaucoma by altering nitric oxide or transforming growth factor-β (TGF-β) signal transduction [18].

A series of prior studies have shown that *CAV1* and *CAV2* are expressed in the eye at the RNA [22,23] and protein [24] levels. We investigated the ocular expression of these genes in the human retina. Immunohistochemical analysis demonstrated that both caveolin 1 and caveolin 2 are expressed throughout the inner retina. Immunoreactivity of caveolin 1 was mainly observed in the ganglion cell layer, consistent with a possible role in glaucoma. Perivascular labeling in the retina was also observed. In prior studies, *CAV1* knockout mice were shown to have vascular permeability defects [25] which also might negatively affect retinal ganglion cell survival. Similar to previous studies in the rat [26], our studies of human retina found immunostaining of small vessels with caveolin 2, as well as Müller and retinal ganglion cells of the inner retina. These findings are largely in accord with a previous study of human melanoma eyes [24]. Futhermore, the caveolins are also expressed in the ciliary muscle, the TM, and Schlemm’s canal, ocular tissues that are involved in IOP regulation and the pathogenesis of glaucoma. Expression in human retina and tissues of the aqueous humor outflow pathway does not prove that either caveolin 1 or caveolin 2 has a role in glaucoma pathogenesis. However, these expression data do provide some support to the hypothesis that variants in *CAV1* or *CAV2* may be the source of glaucoma risk mapped to chromosome 7q31 [18]. Genotype at this locus did not appear to have a clearly recognizable effect on the pattern or intensity of labeling in the retinas of our small series of human donor eyes.

The minor allele of rs4236601 marks the as yet undiscovered POAG risk factor in the chromosome 7q31 locus. However, there is great variability in the minor allele frequency (MAF) of this marker between the populations in the HapMap database and also between populations studied by Thorleifsson et al. [18]. HapMap data shows that Asian populations have low minor allele frequencies of rs4236601 (0%–1.4%), while African populations have high minor allele frequencies (30%–43%) and Hispanic and non-Hispanic Caucasians have intermediate minor allele frequencies (22% and 28%–29%, respectively) [27]. The variable MAF between populations with different racial and ethnic background was also detected in the report by Thorleifsson et al. [18]. Furthermore, the strongest association detected in the 7q31 locus was a result of a relatively small difference in MAF of rs4236601 between POAG patients (28.7%) and control subjects (22.8%) from Iceland [18]. Even smaller differences in MAF between POAG patients and controls were detected in the replication cohorts of this study. The difference in MAF between POAG patients and controls from the UK, Australia, and China was only 0.9%–2.6%, 2.1%–4.7%, and 1.3%–1.6%, respectively [18]. Together these data suggest that the glaucoma risk factor in the chromosome 7q31 region may have a stronger effect in some populations than others.

We set out to determine the role of the chromosome 7q31 risk allele (rs4236601) in our cohort of patients and controls from Iowa. Our study had sufficient power to replicate the association between rs4236601 and POAG reported in the cohort of subjects from Iceland. However, we detected no significant difference (p=0.50) in the MAF of rs4236601 between POAG patients (28.5%) and control subjects (26.9%) from Iowa. Similarly, we detected no significant difference in rs4236601 genotypes between POAG patients and controls from Iowa (p=0.22). Our association study results with rs4236601 (p=0.5) differ considerably from those reported with the Icelandic population in the original report (p=5.0×10^−10^) [18].

There are at least three possibilities that together or individually could explain the disparity between our results and those of the original report. First, is the very large difference in sample size (n=842 from Iowa versus n=36,140 from Iceland). However, this does not seem to be the complete explanation because if the Iowa cohort were to be expanded to the same size as the Iceland cohort (n=36,140) while maintaining the observed allele frequency difference between POAG patients and controls at rs4236601, it would still fail to produce a significant p-value (p=0.078).

Second, it is possible that the difference in association study results might be due in part to an enrollment bias between patients and controls. The large difference in allele frequencies of rs4236601 between ethnic and racial groups in both HapMap and Thorleifsson’s original report suggests the potential for artificially skewed allele frequencies if patient and control populations aren’t well matched. One possible source of such an enrollment bias is the presence of differing frequencies of exfoliation syndrome in patients and controls. In both the current study and the original report [18], attempts were made to exclude subjects with known exfoliation syndrome from the POAG patient cohorts but not from the control cohorts. This specific enrollment bias is likely to be present in all association studies of POAG that use control groups that were not specifically evaluated for exfoliation syndrome. However, it is likely to have a much stronger effect in populations with a higher frequency of exfoliation syndrome, such as cohorts from Iceland. It is possible that some of the difference in our association study results are due to this enrollment bias coupled with the significant differences in the population prevalence of exfoliation syndrome between Iceland (33% [28]) and in the United States (1.6 to 5.0% [29,30]). Since the MAF of rs4236601 varies greatly between populations, associations based on this SNP may be particularly sensitive to ethnic biases.

Finally, the chromosome 7q31 glaucoma risk factor might be more important in some ethnic populations than others due to a founder effect. The minor allele of rs4236601 might be more prevalent among POAG patients in Iceland than in other countries because a larger proportion of Icelandic patients descended from founders with a POAG risk allele in linkage disequilibrium with rs4236601.

Overall, these data and observations imply that a POAG risk allele at the 7q31 locus is not strongly associated with glaucoma in the Iowa population. Future research including more replication studies, the identification of the specific risk allele in the chromosome 7q31 locus, and ultimately, functional studies will clarify the role of this risk factor in the pathogenesis of POAG.
